# Prognostic value of kallikrein-related peptidase 7 (KLK7) mRNA expression in advanced high-grade serous ovarian cancer

**DOI:** 10.1186/s13048-020-00725-5

**Published:** 2020-10-21

**Authors:** Weiwei Gong, Yueyang Liu, Eleftherios P. Diamandis, Marion Kiechle, Holger Bronger, Julia Dorn, Tobias Dreyer, Viktor Magdolen

**Affiliations:** 1 Clinical Research Unit, Department of Obstetrics and Gynecology, Technical University of Munich, Ismaninger Str. 22, D-81675 Munich, Germany; 2 Department of Hematology-Oncology, Guangzhou Women and Children’s Medical Center, Guangzhou, People’s Republic of China; 3 Department of Gynecology, Guangdong Provincial People’s Hospital and Guangdong Academy of Medical Sciences, Guangzhou, People’s Republic of China; 4 Department of Laboratory Medicine and Pathobiology, University of Toronto, Toronto, ON Canada

**Keywords:** Kallikrein-related peptidase, KLK7, Ovarian cancer, Prognostic value, KLK5

## Abstract

**Background:**

High-grade serous ovarian cancer (HGSOC) is the most common and lethal subtype of ovarian cancer. A growing body of evidence suggests tumor-supporting roles of several members of the kallikrein-related peptidase (KLK) family, including KLK5 and KLK7, in this cancer subtype. In normal physiology, KLK5 and KLK7 are the major proteases involved in skin desquamation. Moreover, in several cancer types KLK5 and KLK7 co-expression has been observed. Recently, we have shown that elevated KLK5 mRNA levels are associated with an unfavorable prognosis in HGSOC. Therefore, the aim of this study was to investigate the clinical significance of KLK7 mRNA expression and to explore its relation to KLK5 levels in HGSOC.

**Methods:**

mRNA expression levels of KLK7 were quantified by qPCR in a well-characterized patient cohort afflicted with advanced high-grade serous ovarian cancer (FIGO III/IV, *n* = 139). Previously determined KLK5 mRNA as well as KLK5 and KLK7 antigen concentrations were used to evaluate the relationship between the expression patterns of both factors on the mRNA as well as protein level in tumor tissue of HGSOC patients.

**Results:**

There were strong, significant positive correlations between KLK5 and KLK7 both at the mRNA and the protein level, suggesting coordinate expression of these proteases in HGSOC. In univariate analyses, elevated KLK7 levels as well as the combination of KLK5 + KLK7 (high and/or high versus low/low) were significantly associated with worse progression-free survival (PFS). High mRNA expression levels of KLK7 and the combination of KLK5 and KLK7 showed a trend towards significance for overall survival (OS). In multivariate analyses, KLK7 mRNA expression represented an unfavorable, statistically significant independent predictor for PFS and OS.

**Conclusions:**

The findings imply that both increased KLK5 and KLK7 mRNA expression levels represent unfavorable prognostic biomarkers in advanced high-grade serous ovarian cancer, whereby multivariate analyses indicate that KLK7 mRNA exhibits a stronger predictive value as compared to KLK5 mRNA and the combination of KLK5 and KLK7.

## Background

Ovarian cancer is a heterogeneous disease containing multiple subtypes, with diverse clinicopathologic features and behaviors. High-grade serous ovarian cancer (HGSOC) is the prototype of ovarian neoplasms, representing about 75% of all epithelial ovarian cancer [1, 2]. Due to the lack of obvious symptoms in early stages, most patients are diagnosed at advanced stages and have a high mortality rate [3, 4]. Furthermore, there currently is a lack of accurate, specific biomarkers for ovarian cancer diagnosis and prognosis. CA125 as the most widely used ovarian cancer biomarker is too unspecific and not sensitive enough to serve as a diagnostic tool, especially in early stages. CA125 can be used in therapy monitoring, but fails to predict therapy success. The risk of ovarian malignancy algorithm (ROMA), which includes CA125 and HE4 (human epididymis protein 4), aids to assign patients who present with an adnexal mass into a high-risk versus low-risk group for finding an ovarian malignancy, with good discrimination in premenopausal, but less effective in postmenopausal women [5]. Therefore, new tumor-type specific factors for improving the detection and management of HGSOC are urgently needed.

The kallikrein-related peptidase (KLK) family is the largest contiguous cluster of serine proteases in the human genome, located on the long arm of the human chromosome 19q13.4 and encoding 15 closely related serine proteases [6]. Most KLKs are not only aberrantly expressed in nearly all types of human solid tumors [7, 8], but moreover, have been demonstrated to be involved in tumor growth, migration, invasion, angiogenesis, and chemoresistance [9–11].

In human skin epidermis, several KLKs are coordinately expressed and form an extracellular proteolytic network [6]. Within this proteolytic system, KLK5 and KLK7 are the major proteases predominantly responsible for skin desquamation by directly cleaving corneodesmosomal cadherins [12–14]. In contrast to most other members of the KLK family, which display trypsin-like activity, KLK7 is a chymotrypsin-like serine protease cleaving preferably after phenylalanine, tyrosine and leucine [15–18]. Previous research has demonstrated that KLK7 is implicated in various pathophysiological processes in diverse human organs, such as skin, lung, breast, prostate, and ovary [19, 20]. Especially, in different types of cancer, including breast [21], ovarian [22, 23], pancreatic [24], colorectal [25], and lung tumors [26], expression of KLK7 was shown to be dysregulated.

In ovarian cancer, KLK7 overexpression was found to induce chemoresistance to paclitaxel and increase levels of α5/β1 integrins, which may promote ovarian cancer cell dissemination [23]. In various studies, the prognostic value of KLK7 overexpression, both on the mRNA and the protein level, was analyzed, with conflicting results. On the one hand, elevated KLK7 levels were reported to be associated with poor prognosis on protein [22, 27] and mRNA level [28], whereas, on the other hand, an association of KLK7 with favorable prognosis of ovarian cancer patients has been indicated in other studies [29, 30].

Recently, we have analyzed KLK5 mRNA expression by quantitative polymerase chain reaction (qPCR) in a homogenous cohort encompassing only patients afflicted with advanced high-grade serous ovarian cancer [31]. The results showed that elevated KLK5 mRNA levels predict poor progression-free survival (PFS) of the patients. KLK5 and KLK7 are majorly involved in skin desquamation, a process which may be similar to the shedding of tumor cells from the primary tumor in ovarian cancer leading to intraperitoneal dissemination [32]. Furthermore, KLK5 and KLK7 are co-expressed in different cancer types such as breast cancer or oral squamous cell carcinoma [33, 34]. Therefore, we aimed at exploring the interrelation between KLK5 and KLK7 mRNA levels as well as the clinical relevance of KLK7 expression in the same ovarian cancer patient cohort, which was previously used for KLK5 mRNA analysis.

## Methods

### Patients

Tumor tissue samples of one-hundred and thirty-nine patients afflicted with advanced high-grade ovarian cancer (HGSOC, FIGO III/IV) were included in the retrospective study from the biobank of the Department of Obstetrics and Gynecology and the Institute of Pathology, Klinikum rechts der Isar, TU Munich, Germany, from 1990 to 2012 (for details see [31]). Written informed consent was obtained from all patients.

### KLK5 and KLK7 protein expression levels

KLK5 [35] and KLK7 [29] antigen levels in tumor tissue extracts of 46 of the 139 cases of the present patient cohort have been previously determined by ELISA. ELISA and qPCR analyses of the present project were performed with independent tissue samples of the same patient.

### Quantitative polymerase chain reaction (qPCR)

A detailed description of RNA isolation, reverse transcription, and qPCR has been previously published [36]. Quantification of KLK5 mRNA levels has been described in our previous report [31].

The assay for quantification of KLK7 mRNA is based on the Biosystems TaqMan gene expression assay from ThermoFisher, which consists of a pair of unlabeled PCR primers and a TaqMan probe with a FAM dye label at the 5 ‘end and a minor groove binder as well as a non-fluorescent quencher at the 3’ end (assay ID: Hs00192503_m1). The assay detects all the major transcript variants of KLK7 (NM_005046.3, NM_139277.2, NM_001207053.1, NM_001243126.1), encoding full-length proteins. The assay for the reference gene HPRT1 has been established in-house using the ProbeFinder software and the Universal ProbeLibrary (Roche, Penzberg, Germany) [31, 36].

Standard dilution series were utilized to estimate the efficiency of the KLK7 assay. Threshold cycles (Ct) were employed to calculate the grade of KLKs mRNA expression compared to the respective housekeeping gene HPRT1 by relative quantification using the 2exp-ΔΔCt method.

### Statistical analysis

Analyses were performed using the SPSS statistical analysis software (version 20.0; SPSS Inc., Chicago, IL, USA) (for details see [31, 37]). Based on the quality criteria applied to exclude unassertive results [36], sample numbers in statistical analyses do not always add up to 139. In all statistical tests of this study, differences were considered to be significant if the *p*-value was ≤0.05.

## Results

### Determination of KLK7 mRNA expression levels by qPCR and analysis of their association with KLK5 expression levels and clinical parameters

KLK7 mRNA expression levels were measured in 139 cases of advanced high-grade serous ovarian cancer tissues. KLK7 mRNA expression was in the range of 0 to 953.22 (median: 79.25). In the same patient cohort, KLK5 mRNA levels have been previously determined as well and ranged from 0 to 644.31 (median: 16.87 [31]).

**Fig. 1:**
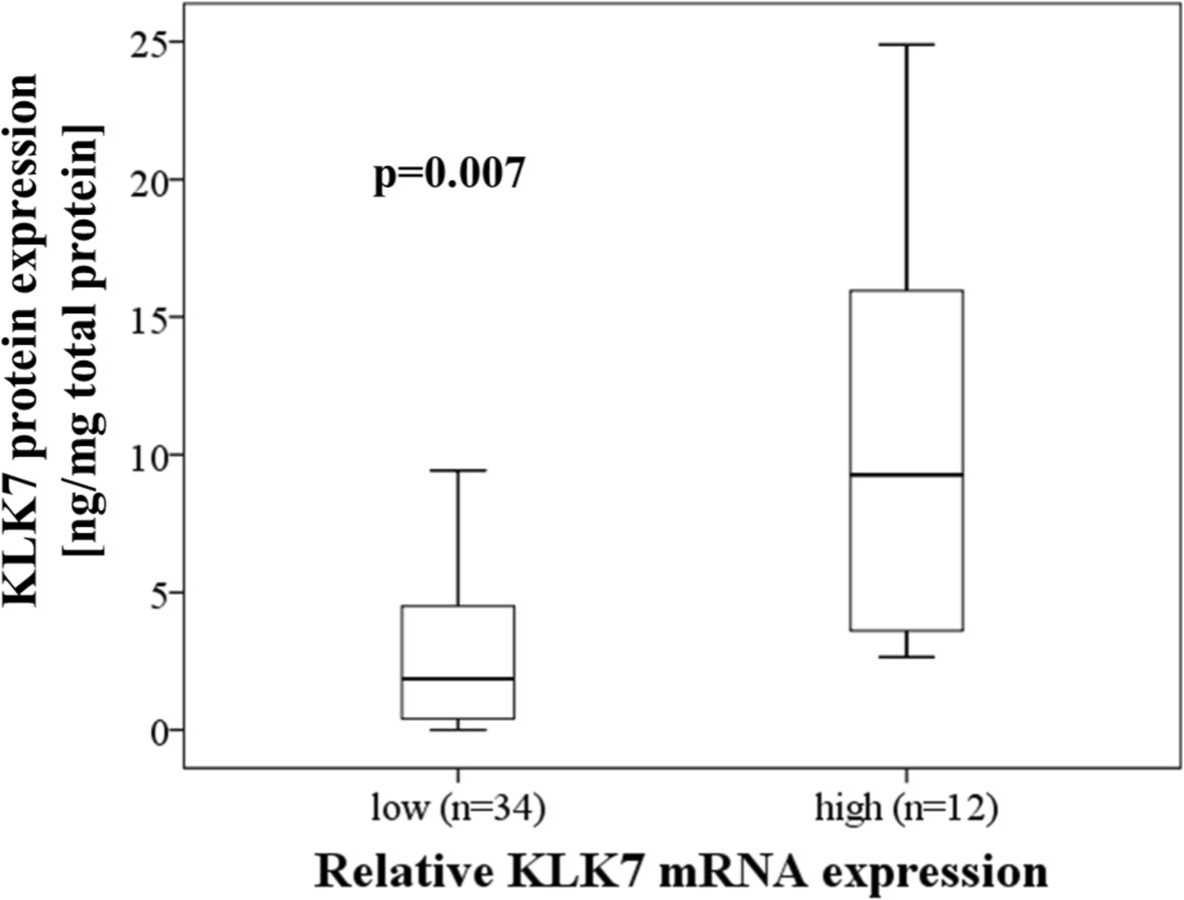
Association of KLK7 mRNA expression levels with KLK7 protein expression in advanced HGSOC. Relative KLK7 mRNA expression levels (normalized to the housekeeping gene HPRT1) were quantified by qPCR and protein expression levels were measured by ELISA (ng KLK7 per mg of total protein, determined in tumor tissue extracts). Higher KLK7 protein levels are present in the group with increased KLK7 mRNA levels (Mann-Whitney test; *p* = 0.007). The 67th percentile (tertiles 1 + 2 versus tertile 3) of the whole patient cohort (*n* = 139) was used as cut-off point for dichotomization of KLK7 mRNA levels into low versus high

KLK7 protein expression data, i.e. antigen levels determined by ELISA in tumor tissue extracts, were available from a previous study analyzing a partially overlapping patient cohort [29]. When correlating KLK7 mRNA expression levels with its corresponding protein expression in the same patients (*n* = 46; Spearman correlation analysis), there was a strong positive correlation between KLK7 mRNA expression and protein expression (r_s_ = 0.663, *p* < 0.001). This finding was validated by box plot analysis (*p* = 0.007; Mann-Whitney test, Fig. 1), where elevated KLK7 protein expression levels are present in the group with higher KLK7 mRNA expression and vice versa. In the above mentioned previous KLK5 study, a similar strong correlation has been observed between KLK5 mRNA and protein levels (r_s_ = 0.689, *p* < 0.001 [31];).

**Fig. 2:**
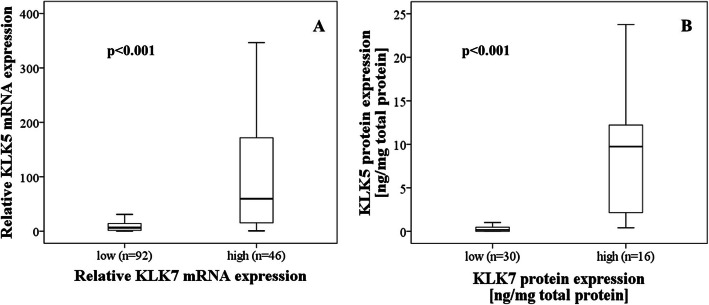
Association of KLK5 expression levels with KLK7 expression levels in advanced HGSOC. By applying the Mann-Whitney test, KLK5 mRNA expression levels were significantly correlated with KLK7 mRNA expression levels (**a**, *p* < 0.001). Similarly, a strong positive relationship was observed between KLK5 and KLK7 at the antigen level, determined by ELISA in tumor extracts (**b**, *p* < 0.001). For both, KLK7 mRNA and antigen levels, the 67th percentile (tertiles 1 + 2 versus tertile 3) was used for dichotomization into a low versus high group

When correlating KLK5 and KLK7, significant associations were observed both at the mRNA levels (r_s_ = 0.568, p < 0.001) as well as the antigen levels (r_s_ = 0.805, p < 0.001). For further analysis, relative mRNA expression levels of KLK5 and KLK7 were grouped by the 67th percentile into a low-expressing (tertiles 1 + 2) versus a high-expressing group (tertile 3) (see [Additional file 1]). Subsequently, the association between KLK5 and KLK7 expression in HGSOC was analyzed by box plot analysis, strongly indicating a coordinate expression of KLK5 with KLK7 in HGSOC (Mann-Whitney test; *p*-value in all cases < 0.001, Fig. 2). Based on this observation, we used a combined factor consisting of KLK5 and KLK7 (KLK5 + KLK7) to further dichotomize the patient cohort into a KLK5 + KLK7 mRNA low-expressing group (KLK mRNA levels below the respective cutoff value) versus a high-expressing group (KLK5 and/or KLK7 mRNA levels above the respective cutoff value) for statistical analyses.

The correlations of KLK7 and KLK5 + KLK7 mRNA expression with established clinical variables, including age, residual tumor mass, and pre-operative ascites fluid volume, are depicted in Table 1.

**Table 1:** Associations of KLK mRNA expression with clinical parameters in advanced high-grade serous ovarian cancer

Clinical parameters	KLK7^**a**^ low/high	KLK5 + KLK7^**b**^ low/high
**Age**	*p* = 0.564	*p* = 0.141
**≤ 60 years**	40/18	26/32
**> 60 years**	56/25	46/34
**Residual tumor mass**	*p* = 0.234	**p = 0.041**
**0 mm**	51/19	43/27
**> 0 mm**	44/23	29/37
**Ascites fluid volume**	*p* = 0.261	*p* = 0.244
**≤ 500 ml**	54/24	45/32
**> 500 ml**	41/13	26/28

^a^ Chi-square test (cut-off point: KLK7 = 67th percentile)

^b^ Chi-square test (dichotomized into low level by KLK5 low and KLK7 low, and high level by KLK5 high and/or KLK7 high; cut-off point: KLK5 = 67th percentile, KLK7 = 67th percentile)

Due to missing values, numbers do not always add up to *n* = 139.

No statistically significant association was found between KLK7 and clinical parameters. The combination of KLK5 + KLK7 mRNA was found to be significantly associated with residual tumor mass (*p* = 0.041), which was previously also observed for KLK5 mRNA alone [31].

### Assessment of the prognostic value of KLK7 mRNA and KLK5 + KLK7 mRNA expression levels in univariate cox regression analysis

By applying univariate Cox regression analysis, the associations of mRNA expression of KLK7, the combination of KLK5 + KLK7 mRNA, and the clinical variables with patient outcome were evaluated in the HGSOC cohort (Table 2). Among the established clinical variables, a positive residual tumor status and a larger ascites fluid volume (> 500 ml) predicted a significantly worse progression-free survival (PFS) and overall survival (OS).

**Table 2:** Univariate Cox regression analysis of KLK mRNA expression levels in advanced high-grade serous ovarian cancer

Clinical parameters	PFS			OS		
	No.^**a**^	HR (95% CI)^**b**^	p	No.^**a**^	HR (95% CI)^**b**^	p
**Age**			0.762			0.414
**≤ 60 years**	43	1		49	1	
**> 60 years**	62	1.08 (0.67–1.72)		70	1.24 (0.74–2.06)	
**Residual tumor mass**			**< 0.001**			**< 0.001**
**0 mm**	58	1		60	1	
**> 0 mm**	47	2.41 (1.53–3.90)		57	3.80 (2.17–6.65)	
**Ascites fluid volume**			**0.019**			**0.005**
**≤ 500 ml**	61	1		66	1	
**> 500 ml**	38	1.79 (1.10–2.90)		46	2.10 (1.25–3.54)	
**KLK7 mRNA**^**c**^			**0.025**			*0.055*
**low**	73	1		82	1	
**high**	32	1.75 (1.07–2.84)		37	1.66 (0.99–2.79)	
**KLK5 + KLK7 mRNA**^**d**^			**0.016**			*0.080*
**low**	52	1		60	1	
**high**	53	1.79 (1.11–2.86)		59	1.58 (0.95–2.63)	

Chi-square test, significant *p*-values (p ≤ 0.05) are indicated in bold, trends towards significance (*p* ≤ 0.08) in italics.

^a^ Number of patients

^b^ HR: hazard ratio (CI: confidence interval) of univariate Cox regression analysis

^c^ Dichotomized into low and high levels by the 67th percentile

^d^ Dichotomized into low by KLK5 low and KLK7 low, and high by KLK5 high and/or KLK7 high; cut-off point: KLK5 = 67th percentile, KLK7 = 67th percentile

Due to missing values, numbers do not always add up to *n* = 105 (PFS) and *n* = 119 (OS).

KLK7 mRNA levels were a significant unfavorable predictor for PFS (HR = 1.75, *p* = 0.025) and showed a trend towards significance for OS (HR = 1.66, *p* = 0.055). The prognostic strength of KLK7 mRNA expression concerning PFS was validated using the online tool Kaplan-Meier Plotter - Ovarian Cancer (HR = 1.31, *p* = 0.034; for details see [Additional file 2]). The prognostic value of the combined factor was found to be mildly increased for PFS (HR = 1.79, *p* = 0.016). Similar to KLK7 mRNA, elevated KLK5 mRNA levels were previously observed to be significantly connected with shortened PFS (HR = 1.60, *p* = 0.047), but not with OS [31].

**Fig. 3:**
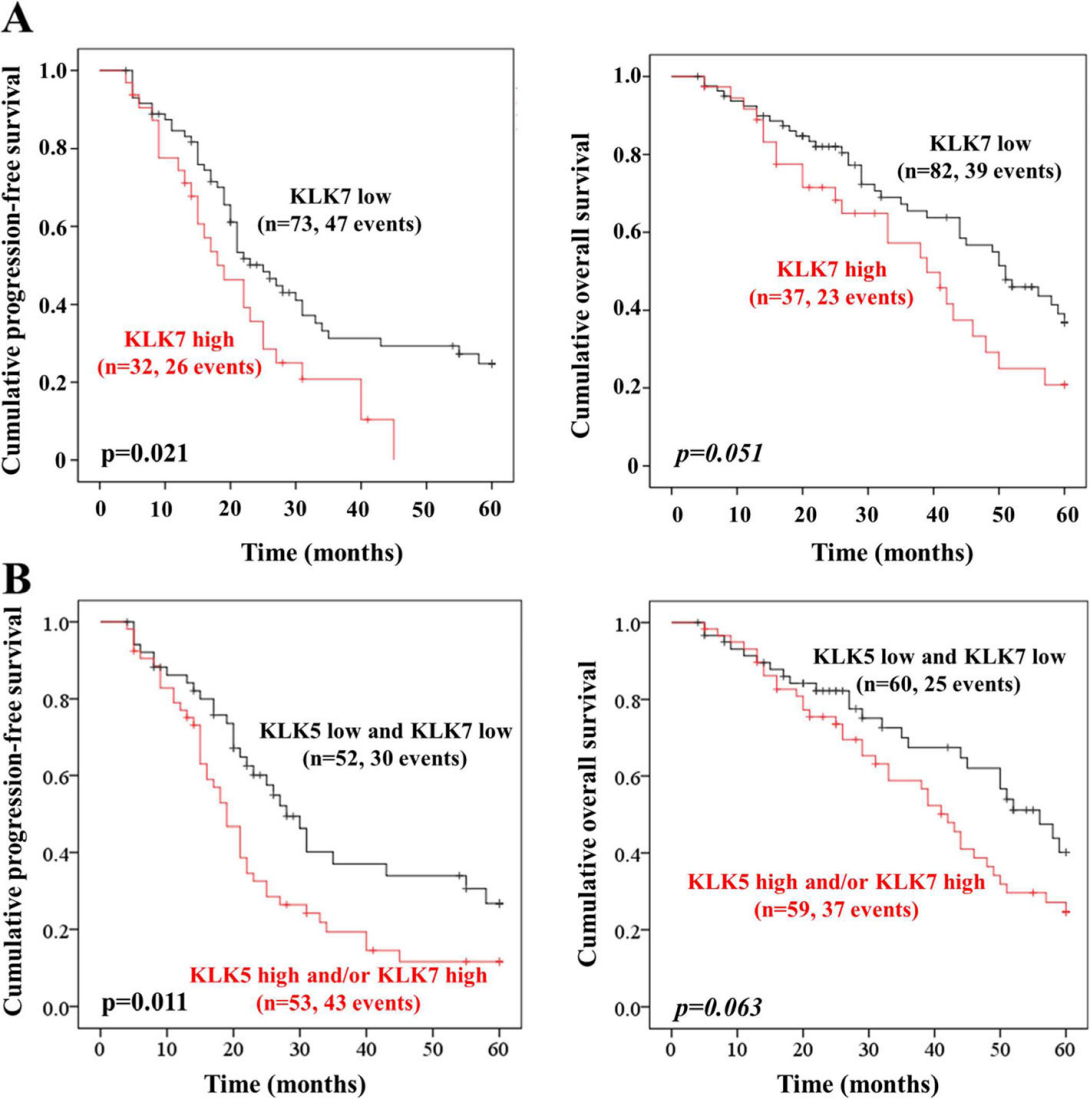
Kaplan–Meier survival analysis concerning KLK7 mRNA expression as well as KLK5 + KLK7 mRNA expression in advanced HGSOC. **a** Patients with elevated KLK7 mRNA expression levels have a significantly worse PFS (*p* = 0.021), compared to those with low mRNA expression levels. **b** The combined factor KLK5 + KLK7 mRNA also represents a prognostic factor for PFS (*p* = 0.011). Both factors show a trend towards significance for OS

The prognostic value of KLK7 mRNA levels as well as of the combination of KLK5 + KLK7 mRNA was further validated by Kaplan-Meier survival analysis. High KLK7 (Fig. 3a) as well as the combined factor (Fig. 3b) displayed a notable correlation with shortened PFS (*p* = 0.021, *p* = 0.011, respectively) and both showed a trend towards significance for OS.

### Assessment of prognostic values of KLK7 mRNA and KLK5 + KLK7 mRNA expression levels in multivariate cox regression analysis

The independent prognostic value of KLK7 mRNA as well as of the combination of KLK5 + KLK7 mRNA in HGSOC was further determined by applying multivariate Cox regression analysis (Table 3). A base model, including age, residual tumor mass, and ascites fluid volume, was constructed for further examination. Here, residual tumor mass was the only clinical variable exhibiting predictive value for PFS (*p* = 0.005) and OS (*p* < 0.001). When individually added to the base model, elevated KLK7 mRNA levels represented an independent unfavorable predictive factor for both PFS (*p* = 0.010) and OS (*p* = 0.041). The combination of KLK5 + KLK7 mRNA expression levels showed a trend towards significance for PFS (*p* = 0.067), while KLK5 mRNA values alone were demonstrated to lose its prognostic power for PFS in multivariable Cox regression analysis [31].

**Table 3:** Multivariate Cox regression analysis of KLK mRNA expression levels in advanced high-grade serous ovarian cancer

Clinical parameters	PFS			OS		
	No.^**a**^	HR (95% CI)^**b**^	p	No.^**a**^	HR (95% CI)^**b**^	p
**Age**			0.592			0.660
**≤ 60 years**	41	1		46	1	
**> 60 years**	57	0.87 (0.53–1.44)		63	1.13 (0.65–1.96)	
**Residual tumor mass**			**0.005**			**< 0.001**
**0 mm**	57	1		59	1	
**> 0 mm**	41	2.20 (1.27–3.81)		50	3.29 (1.69–6.41)	
**Ascites fluid volume**			0.363			0.605
**≤ 500 ml**	60	1		64	1	
**> 500 ml**	38	1.33 (0.76–2.31)		45	1.18 (0.63–2.20)	
**KLK7 mRNA**^**c**^			**0.010**			**0.041**
**low**	72	1		80	1	
**high**	26	2.14 (1.20–3.82)		29	1.89 (1.03–3.49)	
**KLK5 + KLK7 mRNA**^**d**^			*0.067*			0.313
**low**	51	1		59	1	
**high**	47	1.59 (0.97–2.61)		50	1.33 (0.76–2.31)	

Chi-square test, significant *p*-values (*p* ≤ 0.05) are indicated in bold, trends towards significance (*p* ≤ 0.08) in italics.

^a^Number of patients

^b^*HR* hazard ratio (*CI* confidence interval) of multivariate Cox regression analysis

^c^Dichotomized into low and high levels by the 67th percentile

^d^Dichotomized into low level by KLK5 low and KLK7 low, and high level by KLK5 high and/or KLK7 high; cut-off point: KLK5 = 67th percentile, KLK7 = 67th percentile

## Discussion

In ovarian cancer, both KLK5 [38] and KLK7 [39] expression levels were previously reported to be up-regulated in ovarian neoplasms, compared to benign and/or low malignant potential carcinomas, indicating that they may represent diagnostic factors in this tumor entity. Within our recent project, we observed robust mRNA expression patterns of KLK5 [31] and KLK7 (this study) in the majority of ovarian tumor tissues, which were in line with data from The Cancer Genome Atlas (TCGA) [40]. Together with available antigen expression data overlapping with our mRNA cohort, KLK5 and KLK7 mRNA expression were both significantly and positively associated with their protein expression levels, suggesting that there is no major post-transcriptional regulation of expression.

Besides, we observed pronounced correlations between KLK5 and KLK7 mRNA levels as well as between their antigen levels, providing good evidence for the coordinate expression of KLK5 with KLK7 in ovarian cancer. Interestingly, we found parallel expression of KLK5 and KLK7 in a triple-negative breast cancer cohort as well (r_s_ = 0.735, *p* < 0.001; an additional figure file shows this in more detail [see Additional file 3]**,** W. Gong, V. Magdolen, J. Dorn, unpublished). In line with our observations, coordinate expression of KLK5 and KLK7 mRNA has also been previously reported by Li et al. [41] and Talieri et al. [33] in two different population-based breast cancer cohorts. These observations may imply that the functions of KLK5 and KLK7 are dependent on each other.

Indeed, co-expression of KLK5 and KLK7 has been demonstrated to play a crucial role in physiological and pathological processes: (i) combined expression of KLK5 and KLK7 was first found in skin and there the two proteases majorly contribute to skin desquamation and inflammation [13, 42]; (ii) in breast carcinomas, both KLK5 and KLK7 were found to be regulated by steroid hormones, thereby contributing to carcinogenesis and tumor progression [15, 43]; (iii) in ovarian cancer, both KLK5 and KLK7 are not only abundantly expressed, but have been shown to participate in proteolytic cascades to modulate the transforming growth factor β (TGF-β) pathway and epithelial-mesenchymal transition (EMT) [44], support cell shedding and peritoneal invasion as well as chemoresistance [32]; (iv) moreover, KLKs, including KLK5, can also induce an activity amplification cascade by activating themselves, each other and also zymogen forms of other proteases in tumors [16, 45].

Concerning the potential function of KLK7 in ovarian cancer, Dong et al. [23] reported that KLK7 promotes multicellular aggregates (MCA) and α5/β1 integrin-dependent cell adhesion, which induce ovarian cancer cell invasion, metastasis, and paclitaxel resistance [46, 47], thereby increasing peritoneal dissemination and reinvasion. Furthermore, KLK7 may stimulate tumor cell invasion and metastasis through cleaving extracellular matrix (ECM) proteins [48] and several adhesion molecules, such as desmoglein-1 and desmocollin-1 [49]. Moreover, KLK7 has been reported as an activator of matrix metalloproteinase-9 (MMP-9) in carcinomas [50], thus displaying a key role in processes of tumor cell migration, invasion, and angiogenesis, further implying the involvement of KLK7 in tumor progression.

Regarding its clinical prognostic value, KLK7 has been analyzed in various human solid tumors, including ovarian cancer. An association of elevated KLK7 protein expression with poor patient outcome has been reported by Psyrri and co-workers [27] in an ovarian cancer cohort encompassing 150 tumor specimens. A similar finding was obtained in another ELISA-based study [22], where patients with higher KLK7 antigen levels displayed advanced stages and worse PFS, compared to those with low levels. Furthermore, higher KLK7 mRNA expression was associated with a higher risk of recurrence and death. Finally, increased KLK7 protein levels have been detected in sera of serous ovarian cancer patients compared to healthy controls suggesting it as a liquid biopsy early detection biomarker [51]. Additionally, KLK7 was found to be related to paclitaxel chemoresistance in epithelial ovarian cancer patients [23, 28]. Taken together, these studies strongly suggest that KLK7 overexpression has a tumor-supporting role and contributes to a more aggressive phenotype in ovarian cancer. In line with this, in the present study we observed that in a homogenous HGSOC patient cohort, high KLK7 mRNA expression was significantly correlated with increased risk of recurrence and turned out to be an independent unfavorable prognostic factor for both PFS and OS. It should be noted, however, that there are also two ELISA-based studies indicating that elevated KLK7 protein levels represent a favorable prognostic biomarker [29, 30]. These inconsistent results may, on the one hand, be due to the application of different techniques and/or tools: in the ELISA studies, the antibodies used may detect only specific KLK7 forms, e.g. concerning the extent of glycosylation, whereas other techniques such as qPCR quantify the total mRNA amount. On the other hand, the differing results may be due to the use of a rather heterogeneous ovarian cancer patient cohort encompassing several subtypes of both low and high grade serous ovarian cancer tumors in the two ELISA studies [29, 30].

In addition to KLK7 mRNA alone, we also analyzed whether the combination of KLK5 + KLK7 mRNA expression (low/low versus high and/or high) represents a better predictive value for a worse outcome of HGSOC patients. In a previous study, we have observed that the combination of the coordinately expressed proteases KLK10 and KLK11 leads to better accuracy in terms of prognostic biomarkers in ovarian cancer than either KLK10 or KLK11 alone [52]. In our cohort, the combination of KLK5 + KLK7 mRNA displayed a slightly better discrimination in Kaplan Meier analysis concerning median PFS than KLK7 or KLK5 alone, but it was not significantly associated with prognosis in multivariable analysis. Only KLK7 mRNA expression remained as an independent prognostic factor in multivariate analysis, and thus, seems to be a more valuable prognostic factor in HGSOC as compared to KLK5 or the combination KLK5 + KLK7.

## Conclusions

In HGSOC, KLK5 and KLK7 are coordinately expressed both on the mRNA level and on the protein level. Both elevated KLK5 and KLK7 mRNA expression can be considered as unfavorable prognostic biomarkers in HGSOC, however, only KLK7 mRNA expression was found to be independently associated with PFS and OS, whereas the use of KLK5 in combination with KLK7 as well as KLK5 alone lose their predictive value in multivariable analysis.

## Supplementary information

**Additional file 1.** Relative KLK5 and KLK7 mRNA expression in advanced HGSOC. The cumulative histograms represent relative KLK5 and KLK7 mRNA expression levels (normalized to HPRT mRNA levels) in the analyzed HGSOC patient cohort. The values for KLK5 mRNA are taken from Gong et al. [31]. Most cases displayed robust mRNA expression levels of KLK5 and KLK7. For further analysis, both KLK5 (A) and KLK7 (B) mRNA levels were categorized by the 67th percentile into a low-expressing group (tertiles 1 + 2) versus a high-expressing group (tertile 3).

**Additional file 2. **Validation of a significant association between KLK7 mRNA expression and progression-free survival of patients using publicly available Affymetrix data. For analysis of the prognostic value of KLK7 mRNA expression, the online tool Kaplan-Meier Plotter - Ovarian Cancer [53] was used (probe ID: 239381_at; data set 2015 [*n* = 1648]) applying the following selection criteria for the patients: (i) serous histological type, (ii) advanced stage (FIGO III/IV), (iii) chemotherapy using platinum compounds, and (iv) a follow-up of 5 years. Kaplan-Meier analysis confirmed that elevated KLK7 mRNA is significantly correlated with a shortened PFS (*p* = 0.034). With regard to OS, no significant correlation with KLK7 mRNA expression was observed.

**Additional file 3. **Association between KLK5 and KLK7 mRNA expression in triple-negative breast cancer. In a well-defined homogeneous cohort of patients with triple-negative breast cancer, KLK5 mRNA expression levels were significantly correlated with KLK7 mRNA expression levels (Spearman correlation analysis: *r*_s_ = 0.735, *p* < 0.001; Mann-Whitney test: *p* < 0.001).

## Data Availability

Data are available via the Ethics Committee of the Medical Faculty of the Technical University of Munich, Ismaninger Str. 22, 81675 Munich, Germany, for researchers who meet the criteria for access to confidential data. According to the Bavarian Data Protection Authority (BayLDA) and the General Data Protection Regulation (GDPR), patient-related data will only be made available to third parties after double-pseudonymization, undertaken by the Dept. of Medical Statistics and Epidemiology, Technical University of Munich. The Ethics Committee of the Medical Faculty of the Technical University of Munich can be contacted at ethikkommission@mri.tum.de.

## Abbreviations

CI: Confidence interval
ECM: Extracellular matrix
EMT: Epithelial-mesenchymal transition
HE4: Human epididymis protein 4
HGSOC: High-grade serous ovarian cancer
HR: Hazard ratio
KLK: Kallikrein-related peptidase
MCA: Multicellular aggregates
MMP-9: Matrix metalloproteinase-9
OS: Overall survival
PFS: Progression-free survival
qPCR: Quantitative polymerase chain reaction
ROMA: Risk of ovarian malignancy algorithm
SCCE: Stratum corneum chymotryptic enzyme
SCTE: Stratum corneum tryptic enzyme
TCGA: The Cancer Genome Atlas
TGF-β: Transforming growth factor β
