# Supplementary figures and images for: Prognostic value of kallikrein-related peptidase 7 (KLK7) mRNA expression in advanced high-grade serous ovarian cancer
Source: J Ovarian Res. 2020 Oct 21;13:125. doi: 10.1186/s13048-020-00725-5 (PMC7579813; doi:10.1186/s13048-020-00725-5)

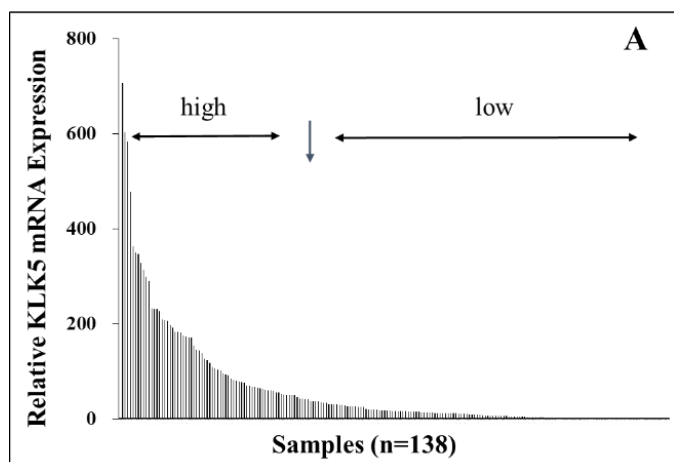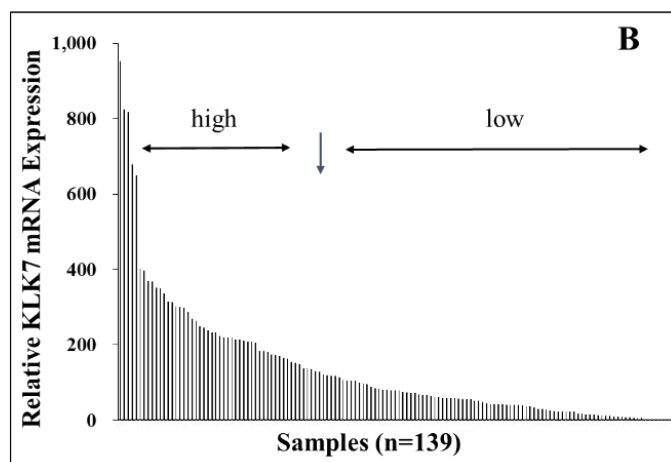

Supplement: Supplementary file 1 — Additional file 1. Relative KLK5 and KLK7 mRNA expression in advanced HGSOC. The cumulative histograms represent relative KLK5 and KLK7 mRNA expression levels (normalized to HPRT mRNA levels) in the analyzed HGSOC patient cohort. The values for KLK5 mRNA are taken from Gong et al. [31]. Most cases displayed robust mRNA expression levels of KLK5 and KLK7. For further analysis, both KLK5 (A) and KLK7 (B) mRNA levels were categorized by the 67th percentile into a low-expressing group (tertiles 1 + 2) versus a high-expressing group (tertile 3). [file 13048_2020_725_MOESM1_ESM.pdf]

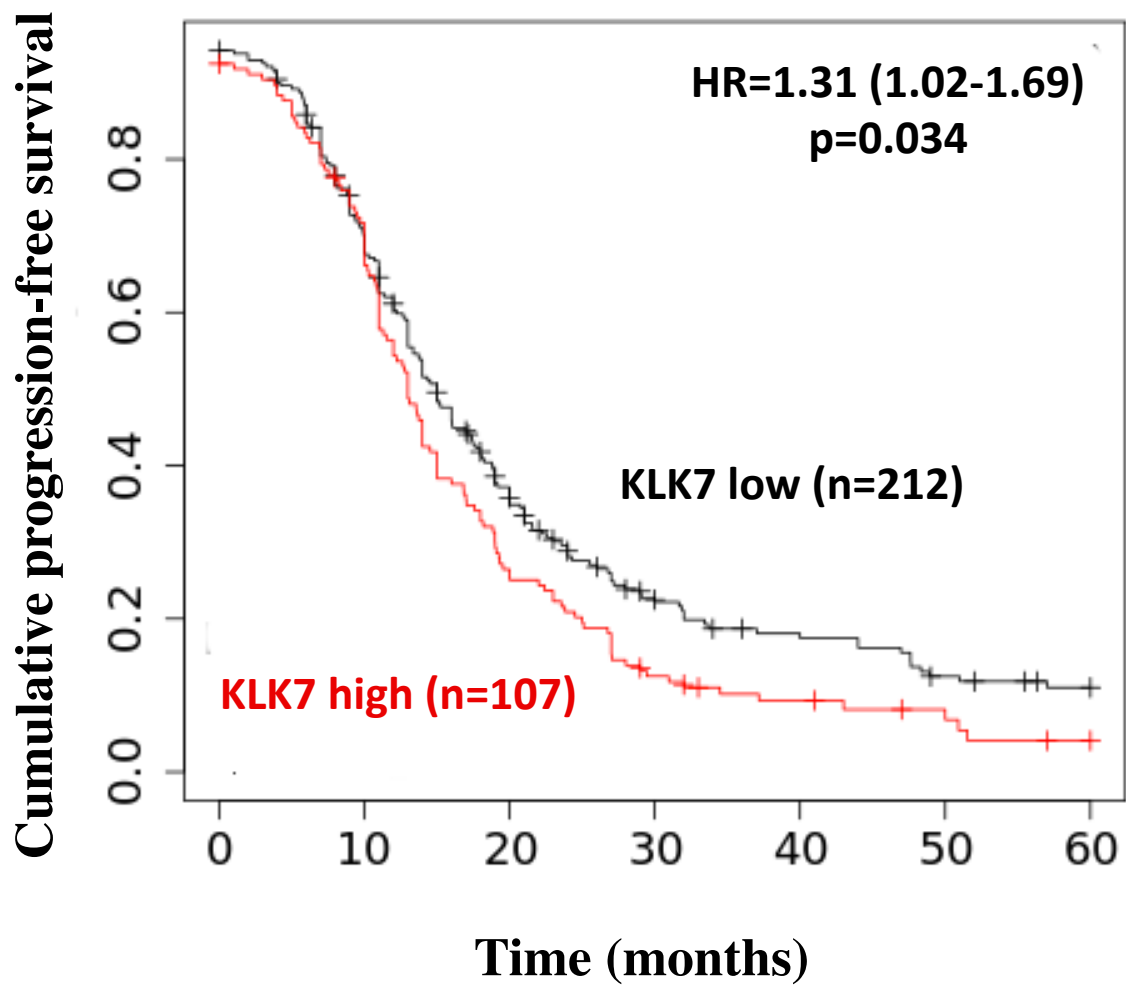

Supplement: Supplementary file 2 — Additional file 2. Validation of a significant association between KLK7 mRNA expression and progression-free survival of patients using publicly available Affymetrix data. For analysis of the prognostic value of KLK7 mRNA expression, the online tool Kaplan-Meier Plotter - Ovarian Cancer [53] was used (probe ID: 239381_at; data set 2015 [n = 1648]) applying the following selection criteria for the patients: (i) serous histological type, (ii) advanced stage (FIGO III/IV), (iii) chemotherapy using platinum compounds, and (iv) a follow-up of 5 years. Kaplan-Meier analysis confirmed that elevated KLK7 mRNA is significantly correlated with a shortened PFS (p = 0.034). With regard to OS, no significant correlation with KLK7 mRNA expression was observed. [file 13048_2020_725_MOESM2_ESM.pdf]

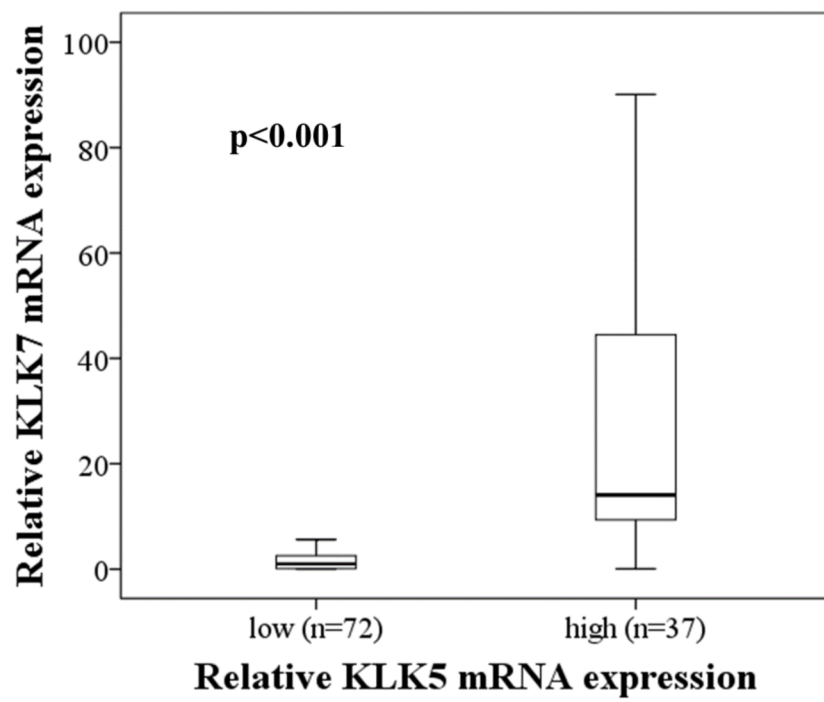

**Additional file 3**

Supplement: Supplementary file 3 — Additional file 3. Association between KLK5 and KLK7 mRNA expression in triple-negative breast cancer. In a well-defined homogeneous cohort of patients with triple-negative breast cancer, KLK5 mRNA expression levels were significantly correlated with KLK7 mRNA expression levels (Spearman correlation analysis: rs = 0.735, p < 0.001; Mann-Whitney test: p < 0.001). [file 13048_2020_725_MOESM3_ESM.pdf]
